# Cross-Modal Object Recognition Is Viewpoint-Independent

**DOI:** 10.1371/journal.pone.0000890

**Published:** 2007-09-12

**Authors:** Simon Lacey, Andrew Peters, K. Sathian

**Affiliations:** 1 Department of Neurology, Emory University, Atlanta, Georgia, United States of America; 2 Department of Rehabilitation Medicine, Emory University, Atlanta, Georgia, United States of America; 3 Department of Psychology, Emory University, Atlanta, Georgia, United States of America; 4 Atlanta Veterans Affairs Medical Center, Rehabilitation Research and Development Center of Excellence, Decatur, Georgia, United States of America; University of Sydney, Australia

## Abstract

**Background:**

Previous research suggests that visual and haptic object recognition are viewpoint-dependent both within- and cross-modally. However, this conclusion may not be generally valid as it was reached using objects oriented along their extended y-axis, resulting in differential surface processing in vision and touch. In the present study, we removed this differential by presenting objects along the z-axis, thus making all object surfaces more equally available to vision and touch.

**Methodology/Principal Findings:**

Participants studied previously unfamiliar objects, in groups of four, using either vision or touch. Subsequently, they performed a four-alternative forced-choice object identification task with the studied objects presented in both unrotated and rotated (180° about the x-, y-, and z-axes) orientations. Rotation impaired within-modal recognition accuracy in both vision and touch, but not cross-modal recognition accuracy. Within-modally, visual recognition accuracy was reduced by rotation about the x- and y-axes more than the z-axis, whilst haptic recognition was equally affected by rotation about all three axes. Cross-modal (but not within-modal) accuracy correlated with spatial (but not object) imagery scores.

**Conclusions/Significance:**

The viewpoint-independence of cross-modal object identification points to its mediation by a high-level abstract representation. The correlation between spatial imagery scores and cross-modal performance suggest that construction of this high-level representation is linked to the ability to perform spatial transformations. Within-modal viewpoint-dependence appears to have a different basis in vision than in touch, possibly due to surface occlusion being important in vision but not touch.

## Introduction

**Figure 1:**
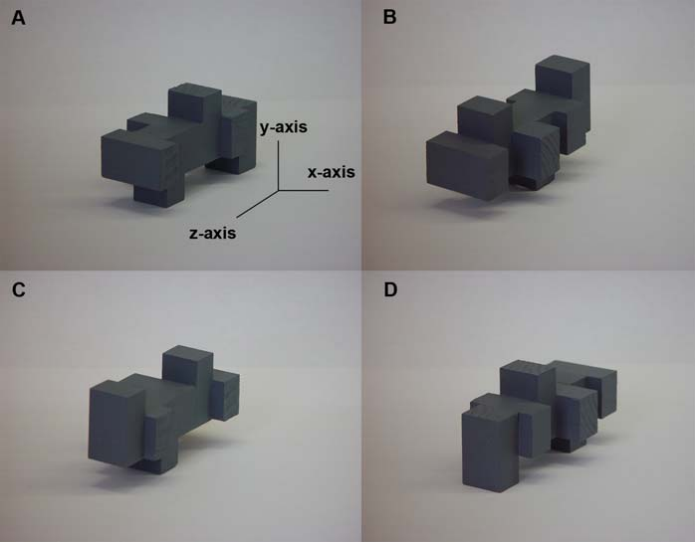
An example object used in the present study in the original orientation (A) and rotated 180° about the z-axis (B), x-axis (C) and y-axis (D).

Previous research suggests that object recognition is viewpoint-dependent within both the visual [1] and haptic [2] modalities, since recognition accuracy is degraded if objects are rotated between encoding and test presentations. However, what happens for visuo-haptic **cross-modal** object recognition is less clear, since differences in the perceptual salience of particular object properties between vision and touch suggest qualitatively different unisensory representations [3], whereas cross-modal priming studies suggest a common representation [4]. *A priori*, one would expect that when touch is involved, representations should be viewpoint-independent because the hands can move freely over the object, collecting information from all surfaces. However, cross-modal recognition was reported to be viewpoint-dependent, improving when objects with an elongated vertical (y-) axis were rotated away from the learned view about the x- and y-axes, and degrading when rotated about the z-axis [2]. The explanation suggested for these findings was that haptic exploration naturally favors the far surface of objects, and vision, the near surface [2]. When objects are rotated about the x- and y-axes, the near and far surfaces are exchanged, the haptic far surface becoming the visual near surface. In contrast, rotation about the z-axis does not involve such a surface exchange. But the haptic preference for the far surface may only be true for objects extended along the y-axis: encoding the near surface of these objects haptically is difficult, given the biomechanical constraints of the hand [2], [5]. If this is true, the observed cross-modal effects might simply reflect the particular experimental design. Here we used multi-part objects extended along the z-axis (Figure 1): this removed the near/far asymmetry since these surfaces were identical facets, making all object surfaces that carried shape information more equally available to haptic exploration. We reasoned that this would allow a truer understanding of the effect of object rotation on cross-modal recognition.

Recognition of rotated objects involves complex mental spatial transformations. In visual within-modal object recognition, mental rotation and recognition of rotated objects have behaviorally similar signatures (in both, errors and latencies increase with angle of rotation) but rely on different neural networks [6]. The relationships between the spatial transformations underlying mental rotation and cross-modal recognition of rotated objects are unclear. As a preliminary step to exploring these relationships further, participants completed the Object-Spatial Imagery Questionnaire (OSIQ) [7] which measures individual preference for both ‘object imagery’ (pictorial object representations primarily concerned with the visual appearance of an object) and ‘spatial imagery’ (abstract spatial representations primarily concerned with the spatial relations between objects, object parts, and complex spatial transformations) [7], [8]. We predicted that performance with our multi-part objects would correlate with the spatial imagery ability reflected in OSIQ-spatial scores, but not with the pictorial imagery ability indexed by OSIQ-object scores.

## Materials and Methods

Forty-eight objects were constructed, each made from six smooth wooden blocks measuring 1.6 cm high, 3.6 cm long and 2.2 cm wide. The resulting objects were 9.5 cm high, the other dimensions varying according to the arrangement of the component blocks. Constructing the objects from smooth wooden component blocks avoided the textural difference between the top and bottom surfaces of Lego™ bricks used by Newell et al. [2]. This was important to obviate undesirable cues to rotation around the x- and y-axes. The objects were painted medium grey to remove visual cues from variations in the natural wood color and grain. Each object had a small (<1 mm) grey pencil dot on one facet that was used to guide presentation of the object by the experimenter to the participant in a particular orientation. Pilot testing showed that participants were never aware of these small dots and debriefing confirmed that this was so in the main experiment also.

The 48 objects were divided into three sets of sixteen, one for each axis of rotation. Each set was further divided into four subsets of four, with one subset for each modality condition. These subsets were checked to ensure that they contained no ‘mirror-image’ pairs. Difference matrices were calculated for the twelve subsets based on the number of differences in the position (three possibilities: in the middle or at either end of the preceding block along the z-axis) and orientation (two possibilities: either the same as, or orthogonal to, the preceding block along the z-axis) of each component block. These values could range from 0 (identical) to 6 (completely different) and were used to calculate the mean difference between objects. The mean difference between objects within a subset ranged from 5.2 to 5.7; the mean of these subset scores within a set was taken as the score for the set and these ranged from 5.4 to 5.5. Paired t-tests on these scores showed no significant differences between subsets or sets (all p values >.05) and the objects were therefore considered equally discriminable.

The procedures were approved by the Institutional Review Board of Emory University. Twenty-four undergraduates (12 male and 12 female, mean age 20 years 3 months) participated after giving informed written consent. Participants performed a four-alternative forced-choice object identification task in two within-modal (visual-visual; haptic-haptic) and two cross-modal (visual-haptic; haptic-visual) conditions. Objects were either unrotated between encoding and test presentations, or rotated by 180° about the x-, y-, and z-axes (Figure 1). In each encoding-recognition sequence, participants learned four objects, identified by numbers, either visually or haptically. Each object was presented for 30 seconds haptically or 15 seconds visually; these times were determined by a pilot experiment. The 2:1 haptic:visual ratio of presentation times reflects that used in previous studies [2], [9], [10]. During visual presentation, participants sat at a table on which the objects were placed. The table was 86 cm high so that the initial viewing distance was 30–40 cm and the initial viewing angle as the participants looked down on the objects was approximately 35–45°. As in the earlier study of Newell et al. [2], the seated participants were free to move their head and eyes when looking at the objects but were not allowed to get up and walk around them.

During haptic presentation, participants felt the objects behind an opaque cloth screen and were free to move their hands around the objects. Unlike the study of Newell et al. [2], the objects were not fixed to a surface but placed in the participants' hands: participants were instructed to keep the objects in exactly the same orientation as presented and not to rotate or otherwise manipulate them. On subsequent recognition trials, the four objects were presented both unrotated and rotated by 180°, about a specific axis from the initial orientation, providing blocks of eight trials. Participants were asked to identify each object by its number. Objects were rotated about each axis in turn, all the modality conditions being completed for a given axis before moving on to the next axis of rotation. The order of the modality conditions, axes of rotation and object sets was fully counterbalanced across subjects.

## Results

**Figure 2:**
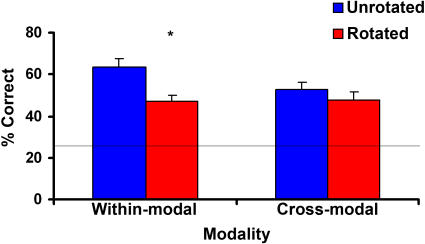
The effect on recognition accuracy of rotating objects away from the learned orientation was confined to the within-modal conditions, with no effect in the cross-modal conditions. (Error bars = s.e.m.; asterisk = significant difference; horizontal line = chance performance at 25% in the four-alternative forced-choice task used).

Figure 2 shows that object rotation substantially degraded recognition accuracy in the within-modal conditions, but only slightly decreased cross-modal recognition accuracy. A two-way (within- vs. cross-modal, unrotated vs. rotated) repeated-measures analysis of variance (RM-ANOVA) showed that object rotation significantly reduced recognition accuracy (F_1,23_ = 30.04, p = <.001) and that overall within-modal recognition accuracy was marginally better than overall cross-modal recognition (F_1,23_ = 4.23, p = .051). These two factors interacted (F_1,23_ = 12.58, p = .002) and post-hoc t-tests showed that this was because within-modal recognition accuracy was highly significantly reduced by rotation (t = 7.25, p <.001) while cross-modal recognition accuracy was not (t = 1.66, p = .11) (Figure 2).

**Figure 3:**
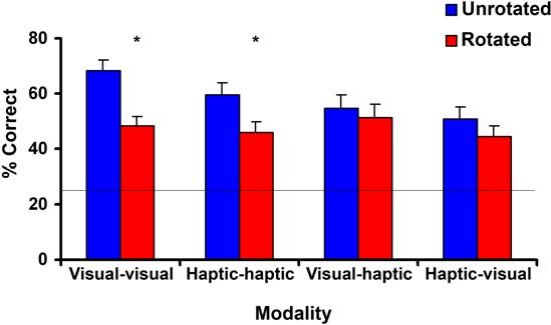
Interaction between modality and rotation. Rotation away from the learned orientation only affected within-modal, not cross-modal, recognition accuracy. (Error bars = s.e.m.; asterisk = significant difference; horizontal line = chance performance at 25% in the four-alternative forced-choice task used).

Analyzing this further, a three-way (modality: within-modal visual, within-modal haptic, cross-modal visual-haptic and cross-modal haptic-visual; rotation; axis) RM-ANOVA again showed a main effect of object rotation (F_1,23_ = 30.04, p = .001) but the axis of rotation was unimportant (F_2,46_ = .39, p = .68), and the main effect of modality fell short of significance (F_3,69_ = 2.49, p = .07). However, modality and rotation again interacted (F_2,46_ = 4.82, p = .004). Three-way (separate within- and cross-modal, rotation, axis) RM-ANOVAs showed again that this was because rotation had an effect in the within-modal conditions (F_1,23_ = 52.57, p <.001) but not the cross-modal conditions (F_1,23_ = 2.74, p = .11). There were no other significant effects or interactions in the cross-modal conditions. Figure 3 illustrates that the two within-modal conditions were similar to each other, as were the two cross-modal conditions.

**Figure 4:**
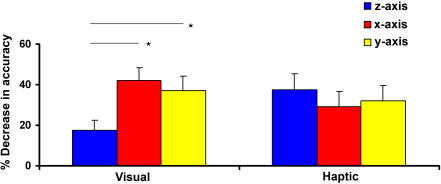
Interaction between the within-modal conditions and the axis of rotation. Haptic within-modal recognition accuracy was equally disrupted by rotation about each axis whereas visual within-modal recognition was disrupted by the x- and y-rotations more than the z-rotation. The graph shows the percentage decrease in accuracy due to rotating the object away from the learned view. (Error bars = s.e.m.; asterisk = significant difference).

In the within-modal conditions, visual and haptic recognition were not significantly different (F_1,23_ = 2.66, p = .12) but modality and axis interacted (F_2,46_ = 4.37, p = .02). To investigate this, we ran separate two-way (axis, rotation) RM-ANOVAs for each modality. While rotation reduced both visual (F_1,23_ = 36.36, p = .001) and haptic (F_1,23_ = 13.54, p = .001) recognition accuracy, there was an effect of axis in vision (F_2,46_ = 3.93, p = .03) but not touch (F_2,46_ = .56, p = .58). To examine this further, we compared the percentage reduction in accuracy for each axis in vision and touch. This was computed using the formula {[unrotated score–rotated score]/unrotated score}*100. (Four observations (2.7% of the total) could not be calculated because the formula required division by zero as there were no correct responses for unrotated objects in these cases; these instances were set to zero). Paired t-tests on these difference scores showed that visual recognition accuracy after z-rotation was significantly better than after x-rotation (t = −2.97, p = .007) or y-rotation (t = −2.19, p = .04): the x- and y-rotations were not different (t = .49, p = .63). In contrast, haptic recognition accuracy was equally disrupted by each axis of rotation (z-x: t = .71, p = .48; z-y: t = .48, p = .63; x-y: t = −.34, p = .73) (Figure 4).

A three-way (rotation, axis, modality) ANOVA of the cross-modal conditions alone showed that there was no main effect of object rotation (F_1,23_ = 2.74, p = .11) or the axis of rotation (F_2,46_ = .03, p = .97), and no significant difference between the two cross-modal conditions (F_1,23_ = 1.34, p = .25). There were no significant interactions.

**Figure 5:**
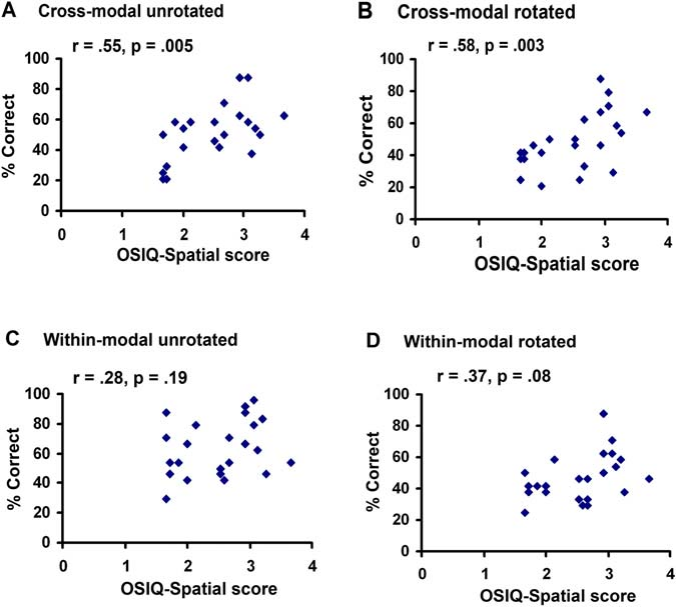
Scatterplots showing that OSIQ-spatial imagery scores correlate with cross-modal (A & B) but not within-modal object recognition accuracy (C & D).

OSIQ-spatial scores were significantly correlated with overall accuracy in both rotated (r = .51, p = .01) and unrotated (r = .48, p = .02) conditions. As Figure 5 shows, OSIQ-spatial scores were also significantly correlated with cross-modal accuracy in both rotated (r = .58, p = .003) and unrotated (r = .55, p = .005) conditions, but not with within-modal accuracy (rotated: r = .37, p = .08; unrotated: r = .28, p = .19). OSIQ-object scores were uncorrelated with accuracy, as predicted.

## Discussion

This study is the first to show that visuo-haptic cross-modal object recognition is essentially viewpoint-independent. Both visual and haptic within-modal recognition were significantly reduced by rotation of the object away from the learned view. This was not so for the two cross-modal conditions. It is well established that, as here, cross-modal recognition comes at a cost compared to within-modal recognition [for example, 11–15], but there was no significant additional cost associated with object rotation. This finding is the more robust because the task in this study was more demanding than in the study of Newell et al. [2] and yet the additional difficulty of object rotation had little effect on cross-modal recognition. For example, although we used similar objects as Newell et al. [2] did (with the exception of the removal of a texture cue) we allowed only half the time for object learning. In addition, participants had to discriminate between specific objects rather than just make a new/old judgment between learned objects and unlearned distractors.

In vision, viewpoint-independence suggests mediation by a high-level, relatively abstract representation [16]. Viewpoint-independence can occur, more trivially, when all object views are familiar [17], perhaps because separate, lower-level representations have been established for each viewpoint; or when the object has very distinctive parts [18] that are easily transformed to match the new viewpoint. However, the objects in the present study were unfamiliar and lacked distinctive parts because the component blocks were identical except in their relationships to one another. Thus, viewpoint-independence could not have arisen simply from object familiarity or distinctiveness of object parts. Rather, the findings of the present study favor the idea of an abstract, high-level, modality-independent representation underlying cross-modal object recognition. Such a representation could be constructed by integrating lower-level, unisensory, viewpoint-dependent representations [16]. Functional neuroimaging studies have demonstrated convergence of visual and haptic shape processing in the intraparietal sulcus (IPS) and the lateral occipital complex (LOC) [19]–[22]. The nature of the representations in these areas is, however, incompletely understood, and has only been studied using visual stimuli. Activity in parts of the IPS scales with the angle of mental rotation [6] and also appears to be viewpoint-dependent [23]. There is a difference of opinion as to whether LOC activity is viewpoint-dependent [24] or viewpoint-independent [23]. Thus, at present, the locus of the modality- and viewpoint-independent, high-level representation underlying cross-modal object recognition is unknown.

The existence of the high-level, modality-independent representation inferred here was obscured in earlier work [2] using objects that were extended along the y-axis. Here, we removed the confounding near-far exchange inherent in this earlier study, by selecting a presentation axis that made all object surfaces more equally available to touch, and demonstrated that cross-modal object recognition is consistently viewpoint-independent across all three axes of rotation. This contrasts with within-modal recognition, where viewpoint-dependence suggests mediation by lower-level, unisensory representations that might feed into the high-level viewpoint-independent representation mediating cross-modal recognition. The correlation between spatial imagery scores and cross-modal, but not within-modal, accuracy, and the lack of any correlation of object imagery scores with performance, suggests that the ability to mentally image complex spatial transformations is linked to viewpoint-independent recognition and supports the view that cross-modal performance is served by an abstract spatial representation.

Our results are also the first to suggest differences between visual and haptic viewpoint-dependence. Rotating an object can occlude a surface and transform the global shape in different ways depending on the axis of rotation [6], suggesting potentially different bases for viewpoint-dependence in vision and touch. Varying the axis of rotation may not matter to touch because the hands are free to move around the object or manipulate it into different orientations relative to the hand. Thus no surface is occluded in touch and it is only necessary to deal with shape transformations. However, these manipulations are not possible visually unless one physically changes location with respect to the object [25], so that vision has to deal with both shape transformations and surface occlusion. Figure 4 suggests that the axis of rotation affects vision but not touch. Visual recognition was best after z-rotation – although this occluded the top surface, the shape transformation is a simple left/right mirror-image in the picture-plane. The x- and y- rotations were more complex; the x-rotation occluded the top surface and produced a mirror-image in the depth-plane. The y-rotation did not occlude a surface but involved two shape transformations, reversing the object from left to right and in the depth-plane. Although it may be counterintuitive that a rotation involving the occlusion of a surface on the main information-bearing axis is easier to process, it should be borne in mind that shape information from the two side surfaces was still available. There is evidence that such picture-plane rotations are easier than depth-plane rotations [6], [26], [27]. Monkey inferotemporal neurons show faster generalization and exhibit larger generalization fields for picture-plane rotations than depth-plane rotations [26]. Face-selective neurons are more sensitive to depth-plane rotations (faces tilted towards/away from the viewer) than to picture-plane rotations (horizontal or inverted faces) [27]. Picture-plane (z-axis) rotations result in faster and more accurate performance than depth-plane (x- and y-axis) rotations in both object recognition and mental rotation tasks, even though these tasks involve distinct neural networks [6]. Thus the picture-plane advantage may be a fairly general one. However, further work is necessary to verify that the differences between vision and touch derive from the nature of shape transformations and the presence of surface occlusion.

Our main conclusion is to clarify an important point about visuo-haptic cross-modal object recognition: that the underlying representation is viewpoint-independent even for unfamiliar objects lacking distinctive local features. Further, despite the unisensory representations each being viewpoint-dependent, there are differences between modalities with the axis of rotation being important in vision but not touch.
